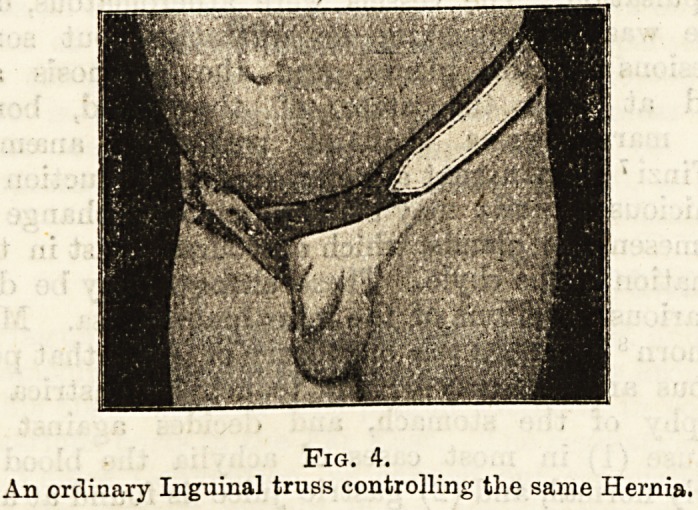# The Incidence and Treatment of Hernia in Young Children.—I

**Published:** 1903-07-04

**Authors:** W. McAdam Eccles

**Affiliations:** Assistant Surgeon St. Bartholomew's Hospital, late Assistant Surgeon City of London Truss Society, and West London Hospital, etc.


### Hospital Clinics and Medical Progress.

### THE INCIDENCE AND TREATMENT OF HERNIA IN YOUNG CHILDREN.—I.

By W. McAdam Eccles, M.S.Lond., F.R.C S.Eng., Assistant Surgeon St. Bartholomew's Hospital, late Assistant Surgeon City of London Truss Society, and West London Hospital, etc.

By some authorities it is said that every case of hernia in young subjects has a congenital origin. Others endeavour to prove that the congenital factor is of little account, and that acquired influences are all important. Neither school of thought is in my opinion correct, and it is necessary to take a combined view of the matter and consider that in most cases at least there is both a congenital and an acquired force at work.

There are only two common forms of hernia in infants and young children, these being the inguinal and the umbilical varieties. It is seldom that a hernia is really present at birth, save in the somewhat rare cases of true congenital umbilical hernia, in which there is in reality a want of development of the anterior abdominal wall in the middle line. But it is nevertheless true that a hernial protrusion may be in evidence but a few days after the entrance of the child into the world.

It is well to remember that during intra-uterine life there is little if any true intra-abdominal pressure, and that the tendency for protrusion of abdominal viscera is therefore almost nil. Further, it may be borne in mind that the contents of the fœtal alimentary tract are aseptic, or at any rate do not contain the bacillus coli communis or its congeners. These micro-organisms, however, put in an appearance before many days in the infant's life have past.

Here then are two factors in the production of hernia which are of the greatest importance, but they are both practically absent at or before birth. Take the instance of an infantile umbilical hernia which appears soon after the fall of the cord. There is no doubt a weak spot at the scar left when the cord has separated, but every child has this, and it is only quite a few comparatively who develop a hernia.

A congenital weakness is present in all, but only the minority have the acquired factor at work which brings about the protrusion. If the infant with this form of umbilical hernia be examined it may be found fat or thin, happy or unhappy, free from cough or suffering from bronchial inflammation, and with or without evidence of obstruction to either the urinary or alimentary apertures. The hernia is an acquired one; what is the essential acquired factor at work? It cannot be merely the weak spot, and it must therefore be sought in the increase in the intra-abdominal pressure which springs into existence soon after the birth of the child.

The chief cause of this heightened pressure is undoubtedly the formation of gas within the intestines, and this gas is in reality due to the action of the bacteria introduced with the food, from the surface of the nipple in the child who is suckled, or from the milk and teat in the child which is hand-fed. The bacillus, as its name expresses, resides and flourishes for the most part in the large intestine, and it is here that the abundance of gas is developed, and because the transverse colon lies so closely in juxta-position behind the site of the weak spot at the scar left by the fall of the cord, that a hernial protrusion is occasioned. Further, a child which has its large intestine distended with flatus is apt to suffer from severe colicky pains, and these induce crying, which further tends to increase the pressure within the abdominal walls, and therefore to force a pouch of peritoneum to protrude.

To sum up, then, it may be stated that the cause of infantile umbilical hernia is almost, if not entirely, acquired rather than congenital—using these expressions in the sense to mean by the former a factor or factors acting after the birth of the child, and by the latter those present before the entrance of the infant into the world. The weak spot in the abdominal wall may be considered of congenital origin, while the increased abdominal pressure due to distension of the intestine, chiefly the colon, with flatus is of a purely acquired character.

Taking the instance of a so called congenital inguinal hernia: here there is a distinctly different state of affairs to that present in the umbilical form. A considerable proportion of children, it is difficult to say approximately how many, but it must be a large number of both male and female infants, are born with a patent processus vaginalis testis or canal of Nuck on one or both sides. This constitutes a truly congenital sac. But here again, although a very large number of infants may possess this pouch of peritoneum, only a small proportion of such cases develop a hernia. Again, an acquired factor must be at work over and above the congenital condition present. It is not far to seek for this. The same factor as in the case of the umbilical form is again potent, but with this interesting difference, that while the navel protrusion often comes into evidence within a few days of the fall of the cord, the inguinal often remains for months or even years before intestine is forced into the waiting sac. The probable reason for this lies in the fact that the mouth of the umbilical protrusion is broad and soon allows the viscera to pouch it, while that of the inguinal pouch is so small, in the majority of cases, that it will require a considerable amount of time to dilate it sufficiently to allow even a loop of empty small intestine to engage in it. It is for this cause also that a strangulated congenital inguinal hernia is usually one in which the nipping is for the time being very acute. Let me here remark in this connection that it is of the utmost importance to examine a child, and particularly a hand-fed child, for the presence or not of a small strangulated hernia when there is vomiting and pain, the cause of which is not too evident. It is disaster to overlook the hernia, and to find it and reduce it is a gratification to all concerned. While I am perfectly willing to admit that there are many other causes of an acquired nature which may be partly responsible for the oncoming of the hernia, yet I am convinced that by far the larger majority of these instances of so-called congenital inguinal herniæ are due to the presence of a congenital sac, and the existence of distended intestine from improper diet.

Take for instance the often stated fact that congenital phimosis induces a hernia. It is possible that it may act as one of the exciting causes in those instances in which there is real obstruction to the outflow of urine, but such instances in my experience are not only comparatively rare, but the cases in which I have seen them have for the most part been those in which no hernial protrusion has been developed. Mere irritation about the prepuce from retained secretion cannot in my opinion be a predisposing cause of hernia, though it may be a serious factor in the production of several forms of nervous conditions. Then again it is a fact that Jewish boys are proportionately more liable to hernia than are others, and it would seem that in them there is a factor of far greater importance, seeing that urinary obstruction is absent.

While commenting upon the question of phimosis, I should like to strongly urge that while phimosis may be a predisposing or even an exciting cause of hernia in some infants, it is altogether a fallacy to believe that circumcision will, without other treatment, bring about the cure of the protrusion. By all means circumcise, this is clearly indicated in most cases, but do not trust to this operation for the cure of the hernia; the protrusion must also be treated by truss or operation. Constipation, bronchitis, and other conditions may also be in evidence, and may be considered to be the cause of the hernia, but in many cases these very affections are the outcome of improper feeding, for they are liable to be associated with signs indicating the presence of rickets, and children suffering from this disease are not infrequently the subjects also of hernia.

Passing now to the treatment of inguinal hernia in children, it may at once be stated that the removal of the exciting cause is all important. Thus a tight foreskin should be ablated, any bronchitis appropriately treated, constipation overcome, and the distension of the abdomen moderated by all the means that are possible. This preliminary and essential part of the treatment having been carried out, it becomes requisite to turn to the actual protrusion itself. This will need one of two methods of treatment. The first is the application and continued wearing of a truss, and the other the radical measure of the removal of the sac by operation.

It is not by any means proper that every case of hernia in young subjects should be submitted to operation. There are several reasons for this statement. One is that many cases of inguinal hernia of the congenital form are cured without operation, another that the child at the time that it is first seen with the hernia is not in a suitable condition for operative proceedings, and yet a third that an operation has a definite risk, which, however, is happily with improved methods becoming less and less every year.

Further, it is not infrequently a wise measure to advise the application of a truss for a time with the object of partially controlling the hernia so as to bring about an improvement in it with a view to a greater certainty of success by an operation performed a few months later. It is extremely rare to meet with a case of so-called congenital inguinal hernia in an infant which cannot be controlled by a suitable truss, provided that the child is at the same time properly dieted.

A truss to be effective in any but the simplest of cases, must have a steel spring, and for infants must be covered with pure black indiarubber for the sake of cleanliness and preservation. Any other form of truss will be unlikely to succeed in bringing about the co-aptation of the walls of the processus vaginalis. It is of the utmost importance that a truss of the right construction, of the proper size, and one that is adjusted in the proper place should be procured, for otherwise failure is sure to follow, and much disappointment, if not actual danger, may result. The measurement for the truss is taken in the usual way by carrying a tape measure obliquely around the pelvis of the child, taking care not to bring the tape lower than the upper border of the symphysis pubis in front. It is common for the measurement to be too large, with the consequence that the truss is too big, and can therefore be placed too low down, in which position it will cause excoriation and be of no use in controlling the protrusion. It is well to remember in this connection that the symphysis pubis in a child is placed at a higher level than is generally supposed. There is, however, a very distinct guide to the level required, for all infants, and

Fig. 1.
An ordinary single rubber-covered Inguinal Truss for an infant. Note the covering of pure black rubber, and the under-strap of lint.

Fig. 2.
An ordinary double rubber-covered Inguinal Truss.

Fig. 3.
A right scrotal hernia in an infant.

particularly those that are well nourished, present a curved line running across the lower part of the abdomen, which accurately indicates the upper limit of the pubic bones. The important fact to be borne in mind is that the whole of the pad of the truss

Fig. 4.
An ordinary Inguinal truss controlling the same Hernia.

must lie above this line, for if it be adjusted so as to lie below it, the soft parts upon which it rests will inevitably be compressed between the pad in front and the unyielding body of the os pubis behind. Very soon such pressure will induce irritation and excoriation, but this untoward result can be entirely avoided if the truss be worn at the proper level, so that the pad only presses upon soft tissues.

A truss thus carefully adjusted must be worn at all times—in bed, when running about, while at ease, and when crying, in the bath and out of it, standing or lying, at night as well as in the day. Further, it should be worn thus for at least three years, and unless there is no obvious bulge at the site of the deep ring after that period, it should either be still worn or an operation advised.

(*To be continued.*)

## Figures and Tables

**Fig. 1. f1:**
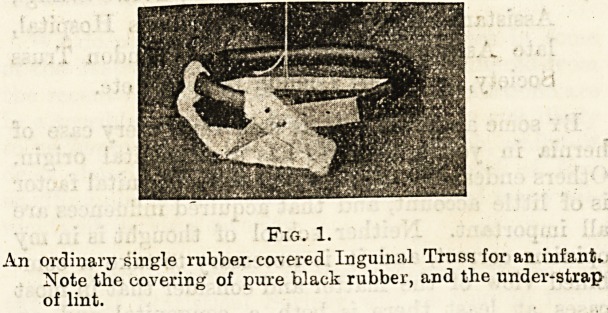


**Fig. 2. f2:**
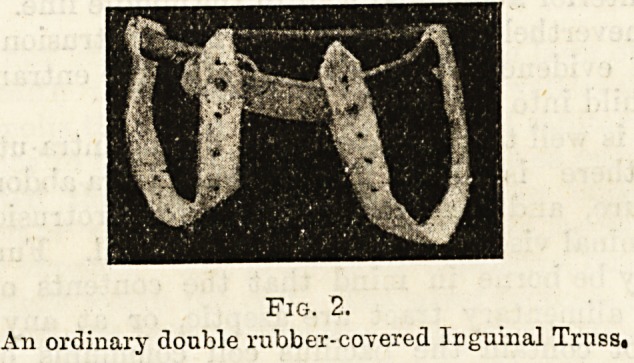


**Fig. 3. f3:**
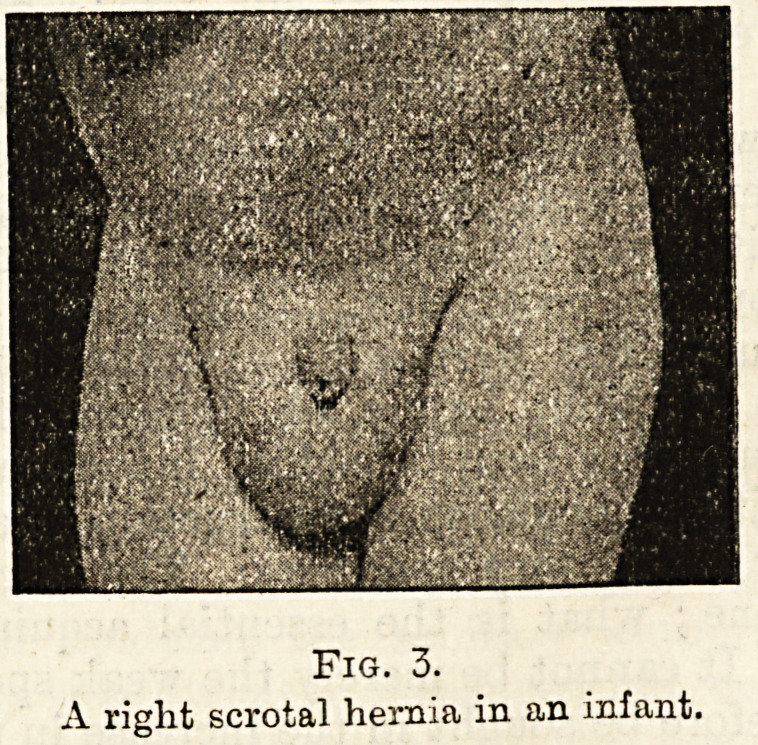


**Fig. 4. f4:**